# SOCS3 treatment prevents the development of alopecia areata by inhibiting CD8+ T cell-mediated autoimmune destruction

**DOI:** 10.18632/oncotarget.16504

**Published:** 2017-03-23

**Authors:** Zhen Gao, Yu-Qing Jin, Wei Wu

**Affiliations:** ^1^ Department of Plastic and Reconstructive Surgery, Shanghai 9th People's Hospital, Shanghai Jiao Tong University School of Medicine, Shanghai, China; ^2^ Department of Plastic Surgery, Shanghai International Medical Center, Shanghai, China

**Keywords:** alopecia areata, SOCS3, IFN-γ, autoimmune, hair follicle

## Abstract

Alopecia areata is one of the most common autoimmune diseases resulting from T cell-mediated damage of hair follicles. CD8+ T cells infiltrate hair follicles and are responsible for destruction of hair follicles. However the underlying mechanisms for hair loss remain still obscure. In the present study, we identified that suppressor of cytokine signaling-3 (SOCS3), a classical inhibitor of cytokine signaling, significantly inhibits CD8+T cell maturation, interferon-γ (IFN-γ) production and alopecia areata. SOCS3 is downregulated in the skin of alopecia areata patients and murine autoimmune alopecia model. Furthermore, SOCS3 treatment prevents the development of alopecia areata in the graft model. SOCS3 decreases the CD44^high^ CD62L^low^ effector memory CD8+ T cells, resulting in the decrease of IFN-γ production. The expression of Fas and major histocompatibility complex-1 (MHC I) is upregulated in skin from C3H/HeJ alopecia areata mice, and this increase is suppressed by SOCS3. The SOCS3 level is negative correlation with the Fas and MHC I level in patients with alopecia areata. These results suggest that SOCS3 treatment may be an effective strategy to treat autoimmune alopecia as well as to more generally prevent cytokine-dependent tissue destruction in inflammatory diseases.

## INTRODUCTION

Alopecia areata (AA) is an autoimmune nonscarring hair loss disorder affecting up to 2% of people worldwide [1]. The hair loss phenotype is usually induced by an inflammatory infiltrate, predominantly comprising CD4+ and CD8+ lymphocytes [2]. Currently, two anti-hair loss drugs, minoxidil and finasteride, have been approved by the FDA [3]. However, new alternative and complementary treatments are required due to their undesirable side effects, low cure rate and high recurrence rate [4].

Anagen HF is one of the immune privilege organs [5, 6]. The collapse of HF immune privilege is considered to the central pathogenic alteration in AA [7]. IFN-γ is a crucial molecule in the pathogenesis of AA [8]. IFN-γ expression is significantly upregulated in AA lesions and contributes to the collapse of HF immune privilege by upregulating MHC I expression in HF [9]. Gilhar *et al* reported that IFN-γ treatment induces follicular expression of MHC I, which results in the collapse of HF immune privilege and induction of autoimmune hair loss in C3H/HeJ mouse model of AA [8, 10]. Additionally, IFN-γ-induced upregulation of chemokines (CXCL9/10/11) and its receptor CXCR3 promotes the accumulation of NKG2D+CD8+ T cells in the skin of AA and induces AA [9]. On the contrary, blockade of IFN-γ markedly inhibits the development of AA in C3H/HeJ mice [11, 12].

Fas/Fas L-mediated apoptosis usually occurs at the development of several autoimmune diseases. Fas expression level is increased on β cells in autoimmune diabetes and upregulated Fas contributes to the pathogenesis of diabetes [13]. Fas/Fas L pathway also plays an important pathogenetic role in AA [14]. Fas expression is increased in skin lesions, and Fas L-neutralizing antibody partially inhibits HF response and hair loss [15].

Cytokines are key modulators of T cell biology, whereas their influence can be attenuated by suppressors of cytokine signaling (SOCS). SOCS proteins regulate cytokine signals that control the differentiation of CD4+ T cells and the maturation of CD8+ T cells from naïve to effector memory states [16]. The role of SOCS proteins in regulating the progression of autoimmune diseases is gradually being revealed. Chong *et al* demonstrated that overexpression of SOCS3 contributes to protect pancreatic β cells from CD8+ T cell-mediated autoimmune destruction [17]. Therefore, SOCS proteins may be a useful strategy for rendering HF unresponsive to multiple cytokines. In the current study we investigate the ability of SOCS3 to prevent the development of autoimmune alopecia.

## RESULTS

### SOCS3 level is downregulated in human and mouse with AA

**Figure 1:**
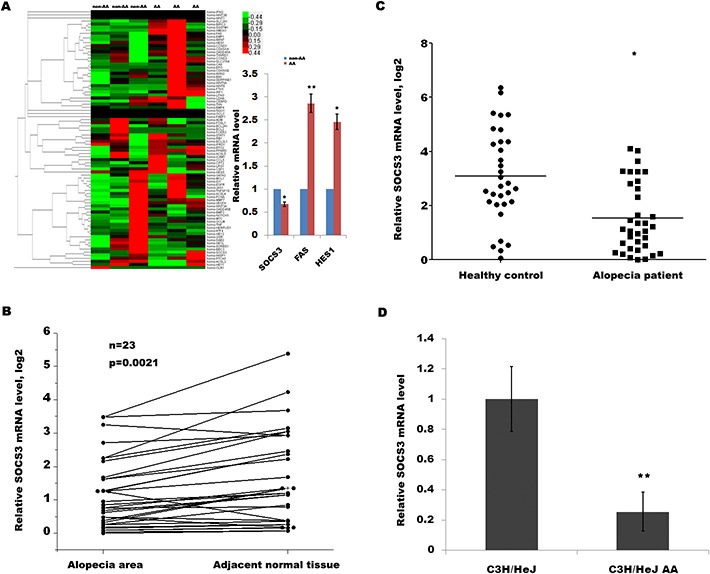
SOCS3 level is downregulated in human and mouse with AA **(A)** Heat map representation of qPCR array data about the expression levels of 84 genes involved in AA progression. Total RNA was extracted from human HF homogenates using Trizol reagent. The reverse transcription for mRNA was carried out using cDNA conversion kit. QPCR for assaying 84 genes expression was performed using a standard protocol from the SYBR Green Real-Time PCR Mix. The GAPDH expression was used as an internal control. Among them, the expression levels of SOCS3, Fas and Hes1 are significantly dysregulated in human AA tissues. *p < 0.05. **p < 0.01. **(B)** qPCR analysis of SOCS3 level in 24 human AA tissues and pair non-AA tissues. The SOCS3 transcripts were expressed at lower levels in most AA tissues (21/24) compared with the non-AA tissues of the same donor. **(C)** qPCR analysis of SOCS3 level in 35 skin tissues of human AA and 32 healthy control. The data showed a significantly lower SOCS3 expression in skin tissues of AA patients than healthy skin tissues. **(D)** qPCR analysis of SOCS3 level in C3H/HeJ AA mice and C3H/HeJ control mice (n = 9). SOCS3 mRNAs were lowly expressed in alopecic skin of C3H/HeJ AA mice compared with control mice. *p < 0.05. **p < 0.01.

AA is a T cell-mediated autoimmune disease of the HF and that cytokines play an important role in the onset of AA partly by inducing apoptosis of HF. To identify key cytokine or signaling pathways involved in AA development, we used a customized qPCR array to characterize the transcriptional landscape of alopecic lesional skin in humans with AA. The qPCR array assays 84 genes including Wnt signaling pathway, apoptosis-regulated signaling, JAK/STAT pathway and cytokines (Supplementary Table 1). As shown in Figure 1A, the expression level of SOCS3, Fas and Hes1 in alopecic lesional skin was significantly dysregulated compared with adjacent normal tissues. The SOCS3 protein can trigger a negative feedback process for overactivated cytokine signaling, which is thought to induce autoimmune diseases. Therefore we focused on SOCS3 and investigated its expression pattern and biological function in AA.

To further verify that SOCS3 is downregulated in patients with AA, we assayed its expression in another panel of patients with AA. The SOCS3 transcripts are expressed at lower levels in most AA tissues (21/24) compared with the non-AA tissues of the same donor (Figure 1B). In an addition, we also compared the expression level of SOCS3 between AA patients and healthy control. Figure 1C shows a significantly lower SOCS3 expression in the skin tissues of AA patients than healthy skin tissues. Consistent with above studies, qPCR data also showed that SOCS3 mRNAs are lowly expressed in the alopecic skin of C3H/HeJ AA mice compared with control mice (Figure 1D). These results indicate a potential role of SOCS3 in the regulation of AA development.

### SOCS3 treatment prevents onset of alopecia in AA skin-grafted C3H/HeJ mice

**Figure 2:**
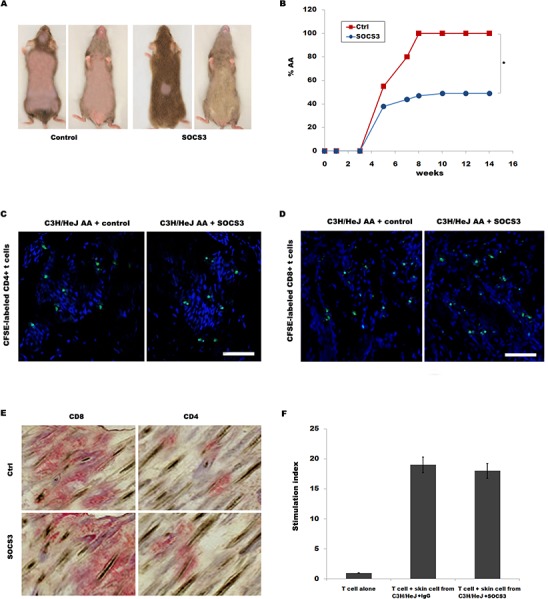
SOCS3 treatment markedly prevents onset of alopecia in AA skin-grafted C3H/HeJ mice C3H/HeJ mice that spontaneously developed AA were euthanized, and full thickness skin grafts were removed and grafted to C3H/HeJ mice. Mice then were treated beginning the day of grafting (n = 9 mice/group). Recombinant mouse SOCS3 protein or IgG control was administrated by i.p. injection (250 μg) two times weekly for 14 weeks. **(A)** The onset of alopecia was inhibited by treatment of SOCS3. **(B)** Time course of onset of AA in SOCS3–treated mice and control mice was shown as weeks after grafting. **(C and D)** Migration of CFSE-labeled CD4+ / CD8+T cells into AA skin grafts was evaluated by immunofluorescent microscopy. The results are representative of five independent experiments. **(E)** Immunohistochemical staining of skin biopsies for assaying CD4 and CD8 expression in skin of mice treated with SOCS3 or IgG control. The results are representative of seven independent experiments. **(F)** The ability of SOCS3-treated skin cells to stimulate purified T cell proliferation was assayed *in vitro*. Scale bars =100 μm. *p < 0.05.

We then tested the biological effect of SOCS3 on disease development in skin-grafted mice. C3H/HeJ mice were grafted with AA skin from a C3H/HeJ AA donor, and then intraperitoneally injected with 250 μg of SOCS3 or IgG control two times weekly for 14 week. All mice developed AA by 14 week after grafting in control group, whereas only 49% of mice receiving SOCS3 developed AA (Figure 2A and 2B).

The mononuclear infiltrate in AA is composed of both CD4+ and CD8+ T lymphocytes [18]. We next investigated whether SOCS3 inhibits AA onset by regulating CD4+ or CD8+ T cell migration or proliferation. To determine whether SOCS3 plays a inhibitory role in CD4+ or CD8+ T cell migration into AA alopecic skin, CD4+ or CD8+ T cells were isolated, and after CFSE-labeling, intravenously injected into recipient C3H/HeJ mice within 2-3 week after grafting as previously described [9]. Figure 2C and 2D showed that SOCS3 treatment cannot inhibit migration of CD4+ or CD8+ T cells to inflammatory sites. SOCS3 also did not suppress CD4+ or CD8+ T infiltration in the skin (Figure 2E). To address the direct interaction between skin cells and T cells, the ability of SOCS3-treated skin cells to stimulate purified T cell proliferation was assayed *in vitro*. Figure 2F showed that SOCS3 treatment cannot suppress T cell proliferation compared with IgG control in co-culture of skin cells and T cells. These data suggest that although SOCS3 prevents AA development, SOCS3 do not inhibit T cell migration and infiltrate.

### SOCS3 treatment decreases CD44^high^ CD62L^low^ effector memory CD8+ T cells, resulting in the reduction of IFN-γ production

**Figure 3:**
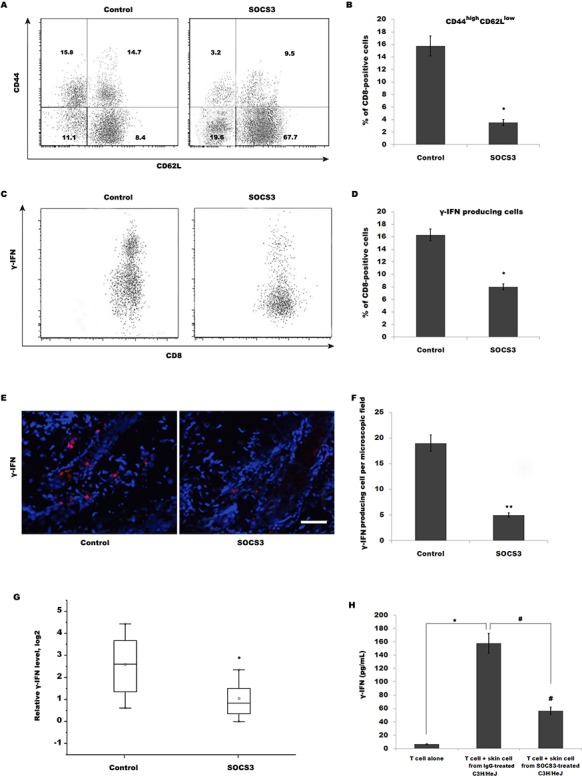
SOCS3 treatment decreases CD44^high^ CD62L^low^ effector memory CD8+ T cells, resulting in the reduction of IFN-γ production **(A)** CD8+ T cells were isolated from SDLNs of SOCS3-treated C3H/HeJ AA mice and were used to assay the level of CD44 and CD62L. Flow cytometry data showed that 15.8% of CD8+ T cells from SDLNs were CD44^high^ CD62L^low^ in C3H/HeJ AA mice, whereas SOCS3 treatment markedly decreases CD44^high^ CD62L^low^ effector memory CD8+ T cells. Data are representative of three independent experiments. Isotype-matched, directly conjugated primary Abs were additionally used to confirm Ab specificity for all markers (data not shown). **(B)** Percentage of CD44^high^ CD62L^low^ effector memory CD8+ T cells was quantitated by flow cytometric analysis. Data are mean ± SD of three independent experiments. **(C)** CD8+ T cells were isolated from skins of SOCS3-treated C3H/HeJ AA mice and were used to assay the percentage of IFN-γ-producing CD8+ T cells. **(D)** Percentage of IFN-γ-producing CD8+ T cells was quantitated by flow cytometric analysis. Data are mean ± SD of three independent experiments. **(E and F)** IFN-γ level in SOCS3-treated AA skin was evaluated by immunofluorescent microscopy. Data are mean ± SD of three independent experiments. **(G)** IFN-γ mRNA level in the skin of grafted recipients was assayed after treatment with SOCS3 or IgG control (n = 6 mice/group). SOCS3-treated group possessed a lower ability to produce IFN-γ compared with control. **(H)** ELISA analysis of IFN-γ production. The amount of IFN-γ produced by T cells stimulated with SOCS3-treated skin cells was decreased compared with that of cells stimulated with IgG-treated skin cells. Scale bars =100 μm.*#*p*< 0.05.

The above data demonstrated that SOCS3 effectively inhibits AA, but SOCS3 do not regulate T cell infiltration into inflammatory sites. Therefore, we speculated whether SOCS3 regulates T cell maturation and its biological function. To characterize the role of SOCS3 in regulating T cell biology in C3H/HeJ AA mice, the expression of molecules associated with T cell maturation, such as CD44 and CD62L, on CD8+ T cells isolated from SDLNs was assayed using flow cytometry analysis. Flow cytometry data showed that 15.8% of CD8+ T cells from SDLNs are CD44^high^ CD62L^low^ in C3H/HeJ AA mice, whereas SOCS3 treatment markedly decreases CD44^high^ CD62L^low^ effector memory CD8+ T cells (Figure 3A and 3B). SOCS3 also blocks the accumulation of IFN-γ-producing CD8+ T cells in the skin in the majority of grafted recipients (Figure 3C–3F). We next assayed IFN-γ mRNA level in the skin of grafted recipients treated with SOCS3 or IgG control. Figure 3G showed that SOCS3-treated group possesses a lower ability to produce IFN-γ compared with control. The amount of IFN-γ produced by T cells stimulated with SOCS3-treated skin cells is decreased compared with that of cells stimulated with IgG-treated skin cells (Figure 3H).

### SOCS3 suppresses IFN-γ-induced upregulation of Fas and MHC I

**Figure 4:**
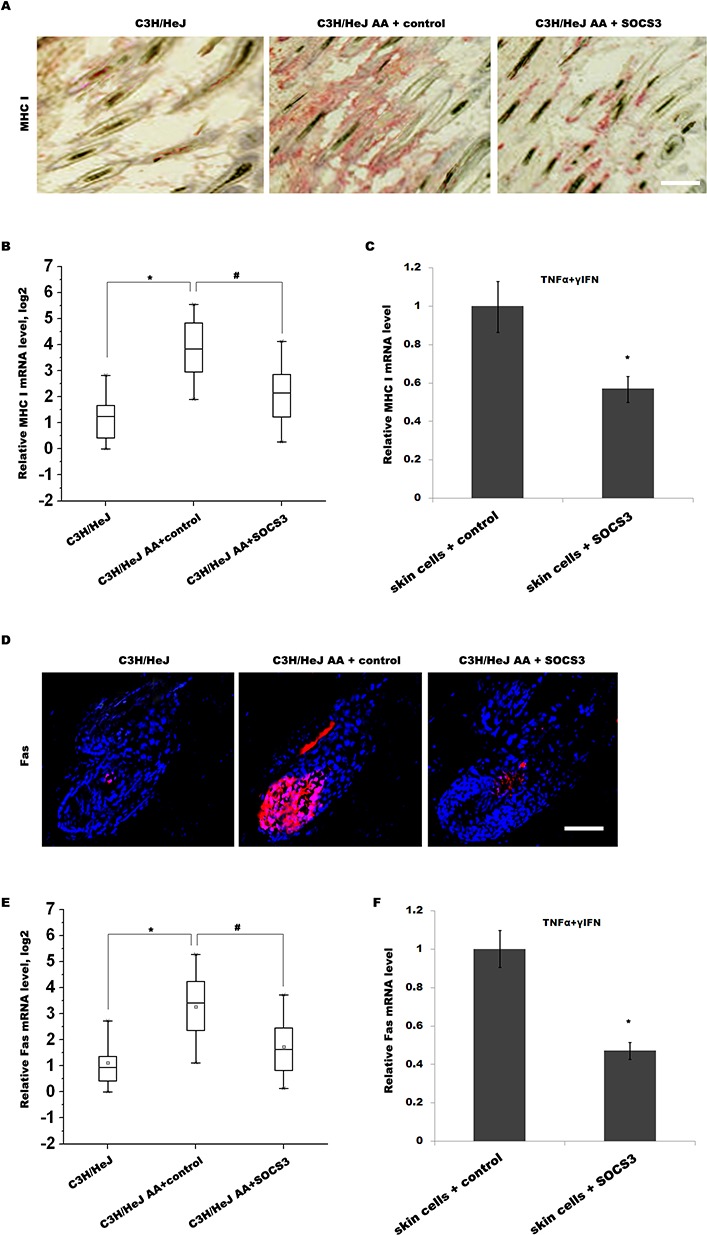
SOCS3 suppresses IFN-γ-induced upregulation of Fas and MHC I **(A)** Immunohistochemical staining for assaying MHC I expression in skin of C3H/HeJ AA mice treated with SOCS3 or IgG control. The expression level of MHC I in C3H/HeJ AA mice was upregulated compared with C3H/HeJ mice, whereas SOCS3 treatment markedly inhibited MHC I expression in C3H/HeJ AA mice. Data are representative of seven independent experiments. **(B)** The MHC I mRNA levels were assayed in skins of C3H/HeJ, C3H/HeJ AA and SOCS3-treated C3H/HeJ AA mice (n = 13 mice/group). **(C)** Skin cells from C3H/HeJ AA mice were treated with TNF-α, IFN-γ and SOCS3, and the expression of MHC I was analyzed using qPCR. TNF-α and IFN-γ upregulated MHC I expression on skin cells, whereas TNF-α/IFN-γ-induced MHC I expression was markedly inhibited by SOCS3 treatment. **(D)** Immunofluorescent staining for assaying Fas expression in skin of C3H/HeJ, C3H/HeJ AA and SOCS3-treated C3H/HeJ AAmice. Data are representative of three independent experiments. **(E)** The Fas mRNA levels were assayed in skins of C3H/HeJ, C3H/HeJ AA and SOCS3-treated C3H/HeJ AA mice (n = 13 mice/group). **(F)** Skin cells from C3H/HeJ AA mice were treated with TNF-α, IFN-γ and SOCS3, and the expression of Fas was analyzed using qPCR. Scale bars =100 μm.*#*p*< 0.05.

IFN-γ is one of the critical factors that lead to the collapse of HF immune privilege and the development of AA [19]. Previous studies showed that IFN-γ promotes the collapse of HF immune privilege by upregulating MHC I expression in the HF [20]. In C3H/HeJ mice, IFN-γ treatment induces follicular expression of MHC I, resulting in the loss of HF immune privilege and the onset of autoimmune hair loss [8]. Thus we next investigated whether SOCS3 inhibits IFN-γ-induced upregulation of MHC I in C3H/HeJ AA mice. As shown in Figure 4A and 4B, the expression level of MHC I in C3H/HeJ AA mice is enhanced compared with C3H/HeJ mice, whereas SOCS3 treatment markedly inhibits the expression of MHC I. The ability of SOCS3 to inhibit cytokine-induced MHC I expression on skin cells was further confirmed *in vitro*. Skin cells were treated with TNF-α and IFN-γ, and the expression of MHC I was analyzed using qPCR. Figure 4C showed that TNF-α and IFN-γ upregulates MHC I expression on skin cells, whereas TNF-α/IFN-γ-induced MHC I expression is markedly inhibited by SOCS3 treatment.

Emerging studies reported that Fas/FasL pathway plays an important pathogenetic role in AA [4, 14]. Fas/FasL-deficient mice are relatively resistant to the induction of AA by grafting of the skin from C3H/HeJ AA mice [14]. In autoimmune type 1 diabetes, IFN-γ-induced upregulation of Fas in pancreatic β cells improves recognition by CD8+ T cells and blocks multiple mechanisms of β cell destruction [17]. Therefore we assayed whether SOCS3 inhibits IFN-γ-induced upregulation of Fas in C3H/HeJ AA mice. As shown in Figure 4D and 4E, the expression level of Fas in C3H/HeJ AA mice is upregulated compared with C3H/HeJ mice, whereas SOCS3 treatment markedly inhibits the expression of Fas in C3H/HeJ AA mice. The ability of SOCS3 to inhibit cytokine-induced Fas expression on skin cells was also confirmed *in vitro*. Skin cells were treated with TNF-α and IFN-γ, and the expression of Fas was analyzed. Figure 4F showed that TNF-α and IFN-γ upregulates Fas expression on skin cells, whereas SOCS3 treatment markedly inhibits TNF-α/IFN-γ-induced Fas expression. These results suggest that SOCS3 prevents AA development by suppressing IFN-γ-induced upregulation of Fas and MHC I.

### SOCS3 prevents AA induced by transfer of CD8+ T cells through inhibiting IFN-γ signaling

**Figure 5:**
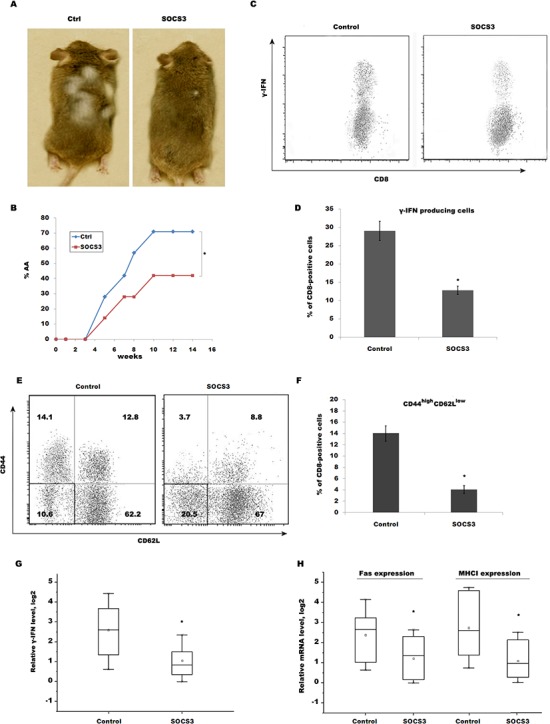
SOCS3 prevents AA induced by transfer of CD8+ T cells through inhibiting IFN-γ signaling CD8+ T cells isolated from SDLNs of AA-affected mice were subcutaneously injected into normal C3H/HeJ recipients. Mice then were subcutaneously injected beginning the day of injection with 250 μg of SOCS3 or IgG (n = 9 mice/group). **(A)** The onset of alopecia was inhibited by treatment of SOCS3. **(B)** Time course of onset of AA in SOCS3–treated mice and control mice was shown as weeks after CD8+ T cells injection. **(C)** CD8+ T cells were isolated from skins of SOCS3-treated C3H/HeJ AA mice and were used to assay the percentage of IFN-γ-producing CD8+ T cells. Data are representative of three independent experiments. **(D)** Percentage of IFN-γ-producing CD8+ T cells was quantitated by flow cytometric analysis. **(E)** CD8+ T cells were isolated from SDLNs of SOCS3-treated C3H/HeJ AA mice and were used to assay the level of CD44 and CD62L. SOCS3 treatment markedly decreases CD44^high^ CD62L^low^ effector memory CD8+ T cells. Data are representative of three independent experiments. **(F)** Percentage of CD44^high^ CD62L^low^ effector memory CD8+ T cells was quantitated by flow cytometric analysis. **(G)** IFN-γ mRNA level in the skin of injected recipients was assayed after treatment with SOCS3 or IgG control (n = 7 mice/group). **(H)** The Fas and MHC I mRNA level in the skin of injected recipients was assayed after treatment with SOCS3 or IgG control (n = 7 mice/group). *p < 0.05.

To further verify that SOCS3 contributes to prevent AA by inhibiting CD8+ T cells maturation and IFN-γ signaling, CD8+ T cells isolated from SDLNs of AA-affected mice were subcutaneously injected into normal C3H/HeJ recipients. 14 week after transfer, ~71% of recipients exhibit patchy, nonscarring hair loss, whereas subcutaneous injection of SOCS3 markedly decreases the occurrence of AA (Figure 5A and 5B). CD8+ T cells isolated from mice skin were then assayed using flow cytometry analysis. Expectedly, SOCS3 treatment blocks the accumulation of IFN-γ-producing CD8+ T cells in the skin of transferring recipients (Figure 5C and 5D). Flow cytometry data showed that 14.1% of CD8+ T cells from IgG-treated group are CD44^high^ CD62L^low^ in transferring recipients, whereas SOCS3 treatment decreases the percentage of CD44^high^ CD62L^low^, indicating that SOCS3 suppresses CD8+ T cell maturation (Figure 5E and 5F). We next assayed IFN-γ level in skin of transferring recipients treated with SOCS3 or IgG control. Figure 5G showed that SOCS3-treated group possesses a lower ability to produce IFN-γ compared with control. SOCS3 treatment further inhibits the expression of Fas and MHC I in skin of transferring recipients (Figure 5H).

**Figure 6:**
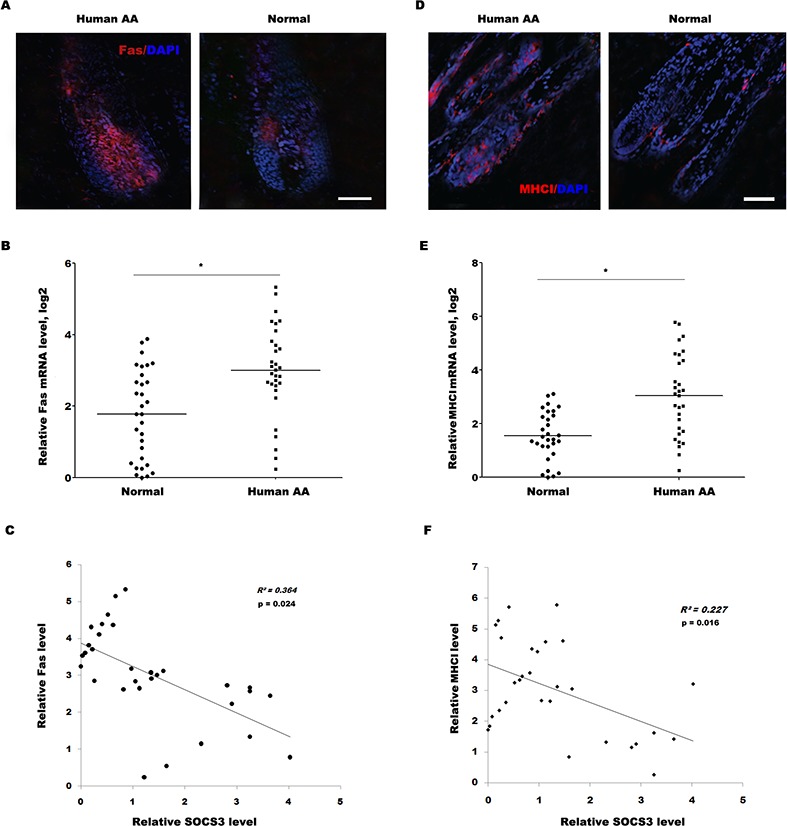
The SOCS3 level is negatively correlated with the Fas and MHC I level in human alopecic skin tissues **(A)** Detection of Fas expression on HF in human AA scalp and healthy control by immunofluorescence analysis. Data are representative of seven independent experiments. **(B)** qPCR analysis of Fas level in 31 skin tissues of human AA and healthy control. The data showed a significantly higher Fas expression in the skin tissues of AA patients than healthy skin tissues. **(C)** The negative association between SOCS3 level and Fas level was measured in 31 human alopecic skin tissues. **(D)** Detection of MHC I expression on HF in human AA scalp and normal control by immunofluorescence analysis. Data are representative of seven independent experiments. **(E)** qPCR analysis of MHC I level in 30 skin tissues of human AA and healthy control. The data showed a significantly higher MHC I expression in the skin tissues of AA patients than healthy skin tissues. **(F)** The negative association between SOCS3 level and MHC I level was measured in 30 human alopecic skin tissues. Scale bars =100 μm. *p< 0.05.

Similarly, the expression level of Fas in most human alopecic skin is remarkably upregulated compared with healthy control as immunofluorescence and qPCR analysis (Figure 6A and 6B). More important, the negative association between SOCS3 level and Fas level was measured in 31 human alopecic skin tissues (Figure 6C). The expression level of MHC I in most human alopecic skin is also upregulated compared with healthy control (Figure 6D and 6E), and the negative association between SOCS3 level and MHC I level was measured in 30 human alopecic skin tissues (Figure 6F). Take together, these data confirm that SOCS3 may be a potentially novel and previously unrecognized target to inhibit AA, a condition for which at the present time there remains no effective therapies.

## DISCUSSION

In this study, we investigated a potential role of SOCS3 in inhibiting AA. The current data demonstrate that (i) SOCS3 level in human and mouse with AA is downregulated, (ii) SOCS3 treatment significantly decreases the occurrence of AA in grafted C3H/HeJ mice, (iii) SOCS3 do not inhibit CD8+ T cell migration and infiltration into inflammatory sites, but reduces CD44^high^ CD62L^low^ effector memory CD8+ T cells, (iv) SOCS3 inhibits IFN-γ signaling, resulting in the downregulation of Fas and MHC I, (v) Finally, we demonstrated that SOCS3 prevents the development of AA by inhibiting CD8+ T cell-mediated autoimmune destruction.

The expression levels of SOCS are low in basal state, but are rapidly induced by the JAK/STAT pathway [21, 22]. SOCS proteins, especially SOCS1 and SOCS3, are often dysregulated in a wide variety of autoimmune diseases [21]. In the study we found that abnormal expression of SOCS3 might cut down the ability to inhibit cytokine production, resulting in the induction of AA. Indeed, intraperitoneal injection of SOCS3 markedly inhibits the occurrence of AA in skin-grafted mice model. Alopecia areata is a T cell–mediated autoimmune disease specific for the hair follicle. After Alopecia areata, naive CD8+ T cells proliferate, produce cytokines, and differentiate into CD44^high^/CD62L^low^ effector memory CD8+ T cells. It is interesting that SOCS3 decreases effector memory CD8+ T cells, given SOCS3 was unable to inhibit T cell proliferation. The result implies that either the mechanisms of T cells proliferation after AA are unaffected by SOCS3 or that remaining mechanisms are sufficient for inducing T cell proliferation. Similarly, our data show that SCOS3 blocks the accumulation of IFNγ-producing CD8+ T cells in the skin, but does not affect CD8+ T cell migration and infiltration into the inflammatory sites. These results also imply that either the mechanisms recruiting T cells to the inflammatory sites are unaffected by SOCS3 or that remaining mechanisms are sufficient for recruitment of T cells.

Previous studies reported that SOCS3 regulates the production of the immunoregulatory cytokines TGF-β1 and IL-10 through modulating STAT3 activation [23]. Yu*et al* demonstrated that SOCS3 deletion in T lymphocytes suppresses development of chronic ocular inflammation via upregulation of CTLA-4 and expansion of regulatory T cells. SOCS3 interacts with CTLA-4 and negatively regulates CTLA-4 levels in T cells, providing a mechanistic explanation for the expansion of regulatory T cells [24]. However our data show that SOCS3 treatment could not significantly block the accumulation of IFNγ-producing CD4+ T cells in skin.

IFN-γ is a critical factor that lead to the collapse of HF immune privilege and the development of AA [19]. In this study, we demonstrated that the MHC I expression in C3H/HeJ AA mice is increased compared with C3H/HeJ mice, whereas SOCS3 markedly inhibits the expression of MHC I in C3H/HeJ AA mice. SOCS3 further suppresses IFN-γ-induced upregulated MHC I on skin cells. Additionally, Fas level in C3H/HeJ AA mice is upregulated compared with C3H/HeJ mice, and SOCS3 treatment decreases the expression of Fas in C3H/HeJ AA mice. The negative association between SOCS3 level and MHC I/Fas level is also detected in human alopecic skin tissues, indicating that the ‘SOCS3-IFN-γ-MHC I/Fas’ pathway is a potential therapeutic target in the improved treatment of AA.

Take together, our data demonstrated that SOCS3 is an important blocker to the development of AA by decreasing CD44^high^ CD62L^low^ effector memory CD8+ T cells. SOCS3 blocks the accumulation of IFN-γ-producing CD8+ T cells in skin, resulting in the decrease of MHC I and Fas expression. In the future, studies targeting SOCS3 and other SOCS proteins may prove useful targets for the treatment of human AA.

## MATERIALS AND METHODS

### Specimens and HF organ culture

Human scalp skin specimens were obtained with written informed consent from adult males undergoing routine hair restoration surgery, adhering to the Helsinki guidelines and following approval by the Institutional Research Ethics Committee of the Shanghai Jiao Tong University School of Medicine. Anagen HFs were microdissected from temporofrontal human scalp skin and cultured within 8 h after surgery [25, 26]. These isolated human scalp HFs in the anagen VI stage of the hair cycle were cultured in William's E medium (12551-032, Invitrogen, Carlsbad, CA) supplemented with 10 μg/mL insulin (Sigma, Hamburg, Germany), 10 ng/mL hydrocortisone (Sigma), 80 IU/mL penicillin, 80 μg/mL streptomycin and 1 mM L-glutamine as previously described [9, 27]. The specimens then were used to cryosection or RNA extraction.

### Mice

C3H/HeJ mice were obtained from the Jackson laboratory (Jackson Laboratories, Bar Harbor, Maine), and maintained in a specific pathogen-free facility on a 12-h light-dark cycle and allowed access to food and water. Transfer of AA was carried out using grafted alopecic C3H/HeJ skin according to the well-established method [2, 9]. Briefly, C3H/HeJ mice that spontaneously developed AA were euthanized, and full thickness skin grafts of approximately 2 cm in diameter were removed and grafted to 6-8 week-old normal-haired female C3H/HeJ mice. Hair loss usually occurred at about 4–6 weeks after grafting.

CD8+ T cells were isolated from SDLNs of AA-affected mice using magnetic bead conjugated monoclonal antibodies and were cultured in RPMI 1640 medium containing 10% FBS (Invitrogen). To induce alopecia, 2 × 10^6^ cells in 100 μl PBS were injected s.c. into the lower right flank of each recipient mouse.

### SOCS3 treatment

Recombinant mouse SOCS3 protein containing a membrane-translocating motif (AAVLLPVLLAAP) was obtained from Cusabio (Wuhan, China) as previous described [28–30] and its bioactivity was verified in our laboratory. Previous studies demonstrated that the recombinant cell-penetrating form of SOCS3 could effectively inhibit the JAK/STAT pathway and attenuate proinflammatory signaling *in vitro* and *in vivo* [28–30]. In the present studies, about 100μl PBS containing 250 μg SOCS3 or IgG control, which was referred to as Jo's study [28] and according to our preliminary results, was administrated by intraperitoneal (i.p.) injection two times weekly for 14 week. Mice were given SOCS3 treatment beginning the day of grafting. Hair status was examined twice weekly and hair growth index was calculated as previously described [11]. Full-thickness skin biopsies were excised from each region of the dorsum at the indicated time, and were used to qPCR or immunohistochemistry analysis.

### Quantitative real-time PCR (qPCR) analysis

Total RNA was extracted from skin or HF homogenates using Trizol reagent (Invitrogen). The reverse transcription (RT) for mRNA was carried out using cDNA conversion kit and the Oligo(dT)18 primer (Invitrogen). QPCR was performed using a standard protocol from the SYBR Green Real-Time PCR Mix (Toyobo, Osaka, Japan) on Applied Biosystems 7300 real-time PCR system (Applied Biosystems, Foster City, CA). The GAPDH expression was used as an internal control. Customized qPCR array was used to assay the expression of 84 genes involved in AA. Primers (forward and reverse listed 5′-3′) used for qPCR and qPCR array were listed in (Supplementary Table 2).

### Single-cell suspensions and flow cytometry

Skin-draining lymphnodes (SDLNs) were dissociated and filtered with a 40-mm cellstrainer. Blood cells were depleted of erythrocytes by ammonium chloride lysis and washed before staining. Flow cytometric analysis of immune cell phenotype was carried out by staining with the following fluorochrome-conjugated Abs: CD8 (12-0081-82, eBioscience, USA), CD44 (16-0441-85, eBioscience) and CD62L (ab25282, Abcam) as described previously [31]. For intracellular IFN-γ assay, 2 × 10^5^ CD8+ T cells were treated with Cell Stimulation Cocktail (eBioscience) for 1 h. Brefeldin A (BD Biosciences) was added for 4 h incubation at 37°C. Cells were then fixed and permeabilized using the Cytofix/Cytoperm kit (BD Biosciences) and stained intracellularly with anti-IFN-γ (XMG1.2, BioLegend) for 30 min at 4°C. The corresponding isotype controls (551954, BD Biosciences) were used for flow cytometry. Data were collected using an LSR II flow cytometer (BD Biosciences). FlowJo 7.6 software is used for the analysis of flow cytometric data.

### Immunohistochemistry

Immunohistochemical staining of formalin-fixed, paraffin-embedded skin sections was carried out using anti-CD4 (clone RM4-5, eBioscience), CD8 (clone 53-6.7, eBioscience) and MHC I (clone H100-5-28, antibodies-online.com) antibodies as previously described [11]. Biotinylated goat anti-rat/mouse IgGs (Abcam) were used as secondary antibody. Streptavidin-HRP staining was assayed using a standard DAB histochemistry kit (Abcam).

### Immunofluorescence staining

Immunofluorescence studies of Fas and IFN-γ skins or HFs were performed using anti-Fas (ab82419, Abcam) and anti-IFN-γ (sc-373727, Santa Cruz Biotech) primary antibodies and corresponding anti-mouse/rabbit secondary antibodies. Nuclei were counterstained with DAPI (Invitrogen). Fluorescence was observed using a FluoView™ FV1000 confocal microscope (Olympus) and images were processed with Photoshop software. Images were post processed using Adobe Photoshop (rotate, crop, brightness, and contrast adjustments only).

### *In vivo* assay of lymphocyte migration

SDLNs-derived CD8+ T cells were enriched from C3H/HeJ AA mice using a CD8+ T cell isolation kit (Miltenyi Biotec, Gladbach, Germany). The purity was > 96%. Lymphocyte migration *in vivo* was assayed using CFSE-labeled T cells as tracers. CFSE (5,6-carboxyfluorescein diacetate succinimidyl ester) is a fluorescent dye that could be used to stably label lymphocytes and track their migration within animals [32]. Briefly, CD8+ T cells were washed with PBS containing 0.1% bovine serum albumin (BSA) (Sigma) and resuspended at a density of 5×10^6^ cells/ml in 0.1% BSA/PBS at a final concentration of 5 μM CFSE (Invitrogen) for 5 min at 37°C, followed by washing with PBS. 1-2 × 10^6^ CFSE-labeled CD8+ T cells were injected i.v. per mouse. Migration of CFSE-labeled T cells into AA skin grafts was evaluated by immunofluorescent microscopy.

### *In vitro* stimulation of T cells with skin cells

Skin single-cell suspensions were prepared as previously described [9]. In brief, mice skin was cut into small pieces and digested it in a solution of RPMI-1640 medium with 2 mg/ml collagenase type 1 (Worthington) at 32°C for 2 h. The digested skin was then minced, passed over 70-mm cell strainer (BD Biosciences), and washed before treatment with SOCS3 or IgG. T cells were purified from SDLNs, and then purified T cells (2 × 10^5^) were stimulated with 1-2 × 10^4^ skin cells treated with SOCS3 or IgG. After 3 days the cells were pulsed with 2.5 *μ*Ci of [^3^H]thymidine for the last 24 h of culture, and proliferation was measured in a [^3^H]thymidine incorporation assay as previously described [17].

2 × 10^5^ skin cells were cultured in RPMI-1640 medium with SOCS3 and meanwhile treated with IFN-γ (50 ng/ml) plus TNF-a (25 ng/ml) for 48 h at 37°C, then the mRNA levels of MHC I and Fas were assayed using qPCR.

### Statistical analysis

The data presented are mean ±standard deviation of at least three independent experiments. Averaged data were compared using unpaired Student's *t* test or one-way ANOVA, followed by the Scheffé test. *P* < 0.05 was deemed statistically significant for all analyses. Statistical analyses were performed using GraphPad Prism or SPSS PASW Statistics v18.0 (SPSS, Inc., Chicago, IL, USA).

## SUPPLEMENTARY MATERIALS TABLES






