# Supplementary material for: SOCS3 treatment prevents the development of alopecia areata by inhibiting CD8+ T cell-mediated autoimmune destruction
Source: Oncotarget. 2017 Mar 23;8(20):33432–43. doi: 10.18632/oncotarget.16504 (PMC5464880; doi:10.18632/oncotarget.16504)
Supplement: Supplementary file 1 [file oncotarget-08-33432-s001.pdf]

SOCS3 treatment prevents the development of alopecia areata by inhibiting CD8+ T cell-mediated autoimmune destruction

SUPPLEMENTARY TABLES

Supplementary Table 1: The qPCR array assays 84 genes involved in AA

See Supplementary File 1

Supplementary Table 2: qPCR primers used in the study

See Supplementary File 2
