# Supplementary material for: SOCS3 treatment prevents the development of alopecia areata by inhibiting CD8+ T cell-mediated autoimmune destruction
Source: Oncotarget. 2017 Mar 23;8(20):33432–43. doi: 10.18632/oncotarget.16504 (PMC5464880; doi:10.18632/oncotarget.16504)
Supplement: Supplementary file 2 [file oncotarget-08-33432-s002.docx]

Supplementary Table.S1. The qPCR array assays 84 genes involved in AA.

| **Sample** | **Genes** |
| --- | --- |
| human | homo-LDHA |
| human | homo-EMP1 |
| human | homo-AXIN2 |
| human | homo-GATA3 |
| human | homo-ACSL4 |
| human | homo-ADM |
| human | homo-ARNT |
| human | homo-CA9 |
| human | homo-CPT2 |
| human | homo-EPO |
| human | homo-SLC2A1 |
| human | homo-BCL2A1 |
| human | homo-ID1 |
| human | homo-WNT1 |
| human | homo-DAB2 |
| human | homo-FOSL1 |
| human | homo-WNT3A |
| human | homo-WNT6 |
| human | homo-STAT1 |
| human | homo-RB1 |
| human | homo-BIRC3 |
| human | homo-FAS |
| human | homo-GADD45A |
| human | homo-MCL1 |
| human | homo-TNF |
| human | homo-TNFSF10 |
| human | homo-IFNG |
| human | homo-SQSTM1 |
| human | homo-MMP7 |
| human | homo-MYC |
| human | homo-WNT2B |
| human | homo-CCL5 |
| human | homo-CCND1 |
| human | homo-CCND2 |
| human | homo-CDKN1A |
| human | homo-EGFR |
| human | homo-GADD45B |
| human | homo-SOCS3 |
| human | homo-BTG2 |
| human | homo-FTH1 |
| human | homo-GCLC |
| human | homo-GCLM |
| human | homo-GSR |
| human | homo-HMOX1 |
| human | homo-NQO1 |
| human | homo-TXN |
| human | homo-TXNRD1 |
| human | homo-BMP2 |
| human | homo-JAG1 |
| human | homo-NOTCH1 |
| human | homo-SERPINE1 |
| human | homo-WNT5A |
| human | homo-PPARD |
| human | homo-WISP1 |
| human | homo-BAX |
| human | homo-BCL2 |
| human | homo-BCL2L1 |
| human | homo-PCNA |
| human | homo-CDKN1B |
| human | homo-VEGFA |
| human | homo-ACSL3 |
| human | homo-ACSL5 |
| human | homo-ATF4 |
| human | homo-BBC3 |
| human | homo-BMP4 |
| human | homo-CEBPD |
| human | homo-CSF1 |
| human | homo-FABP1 |
| human | homo-FCER2 |
| human | homo-HERPUD1 |
| human | homo-HES1 |
| human | homo-HES5 |
| human | homo-HEY1 |
| human | homo-HEY2 |
| human | homo-HEYL |
| human | homo-ICAM1 |
| human | homo-IFRD1 |
| human | homo-IRF1 |
| human | homo-LFNG |
| human | homo-LRG1 |
| human | homo-OLR1 |
| human | homo-PTCH1 |
| human | homo-SLC27A4 |
| human | homo-SORBS1 |
| human | homo-GAPDH |
